# Bifunctional small molecules targeting PD-L1/CXCL12 as dual immunotherapy for cancer treatment

**DOI:** 10.1038/s41392-022-01292-5

**Published:** 2023-03-01

**Authors:** Binbin Cheng, Wei Wang, Ting Liu, Hao Cao, Wei Pan, Yao Xiao, Shuwen Liu, Jianjun Chen

**Affiliations:** 1 School of Medicine, Hubei Polytechnic University, Hubei Key Laboratory for Kidney Disease Pathogenesis and Intervention, Huangshi, 435003 China; 2 School of Pharmaceutical Sciences, Guangdong Provincial Key Laboratory of New Drug Screening, Southern Medical University, Guangzhou, 510515 China; 3 Department of Cardiology, The Sixth Affiliated Hospital, South China University of Technology, Nanhai people’s Hospital, Foshan, Guangdong, 528200 China

**Keywords:** Drug development, Molecular medicine

**Dear Editor**,

Inhibiting PD-1/PD-L1 interaction is a highly promising therapeutic modality.^1^ However, due to the low overall response rate in patients, researchers have attempted to combine PD-L1 inhibitors with other antitumor agents for cancer therapy. Studies have shown that combination immunotherapy of PD-L1 antibodies with CXCL12 inhibitors exhibited synergistic and better antitumor efficacy than monotherapy, indicating the potential clinical utility of targeting both PD-L1 and CXCL12 as dual immunotherapy to treat cancer.^2,3^ However, there are several disadvantages for combination therapy, including unpredictable PK/PD and overlapping toxicities. A potential alternative to combination therapy would be to use a single molecule with dual or multi-targeting capability, as the PK/PD of a single molecule is easily predictable. For example, dual-targeting bispecific antibodies (bsAbs) have gained significant attention in the field of anticancer drug discovery in recent years. Many PD-1/PD-L1-based bsAbs (e.g., anti-PD-L1/TGF-β, anti-PD-1/CTLA-4, and anti-PD-1/LAG-3) have entered clinical trials as dual immunotherapy for treating cancer. However, bsAbs-based dual immunotherapies also suffer from the common drawbacks (e.g., immunogenicity, poor pharmacokinetics) as antibodies, thus it would be of high significance to develop small molecule PD-L1 inhibitor-based dual immunotherapy, as small molecules may overcome the above drawbacks of antibodies.

We have previously reported PD-L1-targeting bifunctional molecules as potential anticancer agents.^4^ To continue our interest in this area, we designed a set of compounds targeting both PD-L1 and CXCL12 simultaneously as potential dual immunotherapy based on the hypothesis that PD-L1 and CXCL12 are two critical biomacromolecules controlling the immunosuppressive tumor microenvironment. Firstly, we analyzed the pharmacophores of PD-L1 inhibitors and CXCL12 inhibitors (Fig. 1a). The tail group of PD-L1 inhibitors and the hydroxyl moiety of CXCL12 inhibitors were exposed to solvent, making them suitable sites for conjugating the two inhibitors via a linker. Thus, twenty-one bifunctional molecules were designed, synthesized (Supplementary Scheme S1), and bioevaluated (Supplementary Table S1).

**Fig. 1:**
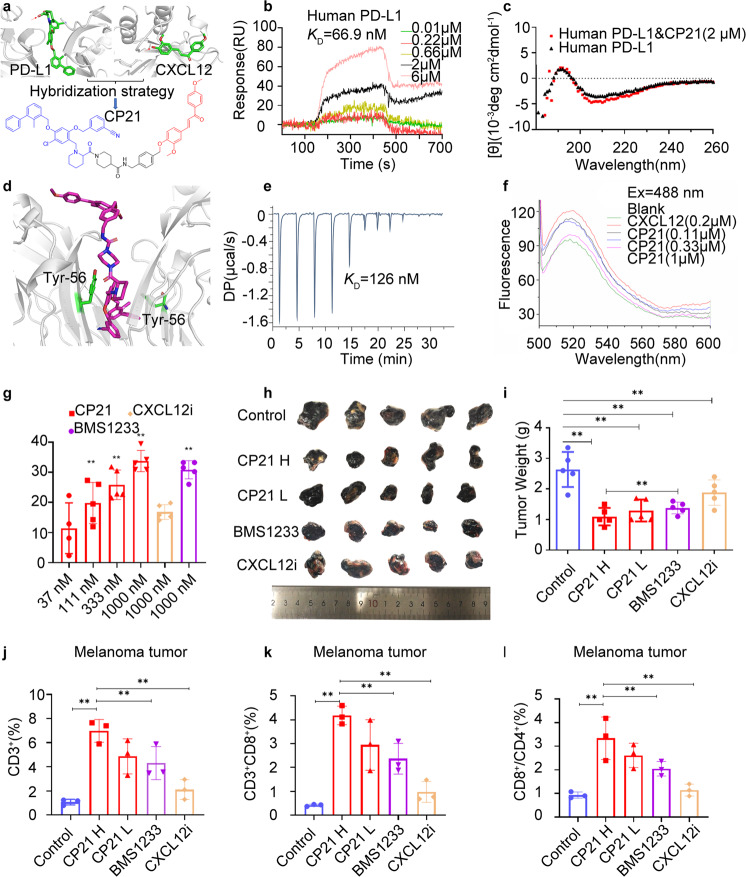
**a** Design of bifunctional molecules targeting PD-L1/CXCL12 based on the pharmacophores of CXCL12 inhibitors and PD-L1 inhibitors. **b** The binding affinity of **CP21** to hPD-L1 as measured by SPR. **c** CD spectra of hPD-L1 in complex with or without **CP21**. **d** Docking of **CP21** to hPD-L1 (PDB code: 6R3K). **e** ITC study for **CP21** in complex with hPD-L1. **f** Intracellular Ca^2+^ release was measured by spectrofluorometry in THP-1 cells. **g** Effects of **CP21** and BMS-1233 (positive control) on HepG2 cell mortality in Jurkat T cell&HepG2 co-culture system (*n* = 5, **P < 0.05). **h** Therapeutic effects of **CP21** (**h**, 125 mg/kg; **l**, 75 mg/kg), BMS-1233, and CXCL12i (inhibitor) against B16-F10 melanoma tumors in mice (*n* = 5). **i** Average tumor weights (**P < 0.05, *n* = 5). Percentages of CD3^+^ cells (**j**) and CD3^+^CD8^+^ cells (**k**), and the ratio of CD8^+^/CD4^+^ cells (**l**) in tumor tissues (*n* = 3, **P < 0.05)

Among them, **CP21** showed the strongest PD-L1-inhibitory effects with IC_50_ of 78.6 nM (HTRF assay). Furthermore, **CP21** displayed similar binding affinity (SPR assay) to both h(human)PD-L1 (*K*_D_ = 66.9 nM, Fig. 1b) and m(mouse)PD-L1 (*K*_D_ = 70.1 nM) (supplementary Fig. S1a). In addition, CD (Circular dichroism) assay revealed that when hPD-L1 or mPD-L1 was mixed with **CP21**, the conformation of their secondary structures changed similarly, as compared to the vehicle which contains only hPD-L1 (Fig. 1c) or mPD-L1 (Supplementary Fig. S1b). Moreover, the microscale thermophoresis (MST) assay confirmed that **CP21** could bind to mPD-L1 with a *K*_D_ of 654.1 nM (Supplementary Fig. S1c, d). Next, the binding affinity of **CP21** to hCXCL12 and mCXCL12 was also determined by SPR and CD. **CP21** bound to hCXCL12 (*K*_D_ = 160 nM, SPR) and mCXCL12 (*K*_D_ = 76.5 nM, SPR) in a dose-dependent manner (supplementary Fig. S1e, f). Furthermore, CD spectroscopy revealed an altered conformation in the secondary structures of hCXCL12 and mCXCL12 upon the addition of **CP21** (Supplementary Fig. S1g, h). The above results suggest that **CP21** could cross-bind to hPD-L1/hCXCL12 or mPD-L1/mCXCL12 proteins with high affinity.

To further investigate the possible binding modes of these dual-acting compounds, we conducted molecular docking analysis for **CP21** with PD-L1 and CXCL12 proteins. As expected, **CP21** fitted nicely in the inner cavity of the PD-L1 dimer (Fig. 1d). Calorimetric data (Fig. 1e and supplementary Fig. S2a) from the ITC assay suggested that the excellent binding affinity of **CP21** to PD-L1 was primarily due to hydrophobic interactions, consistent with molecular modeling studies. Additional molecular docking study also suggested that **CP21** could cross-bind with h/m PD-L1 through different modes (supplementary Fig. S2b–e). Furthermore, **CP21** bound well to the hydrophobic cleft formed by CXCL12 (supplementary Fig. S2f), which was further confirmed by ITC analysis (supplementary Fig. S2g, h). The well-defined molecular modeling studies of **CP21** in PD-L1 and CXCL12 may explain the high binding affinity of **CP21** to its target proteins.

To validate **CP21** as a dual inhibitor of CXCL12 and PD-L1 in vitro, we first evaluated the effects of **CP21** on CXCL12-mediated Ca^2+^ cellular responses in THP-1 cells using a calcium flux assay.^5^ As shown in Fig. 1f, **CP21** dose-dependently inhibited CXCL12-induced calcium responses as measured by spectrofluorometry. Flow cytometry analysis further confirmed that **CP21** abolished the response of CXCL12-mediated Ca^2+^ flux (supplementary Fig. S3a). Moreover, **CP21** dose-dependently inhibited CXCL12-mediated cell migration (supplementary Fig. S3b).

To investigate the immunomodulatory effects of **CP21**, we assessed the mortality rate of HepG2 cells utilizing a Jurkat T&HepG2 cell co-culture model. As illustrated in Fig. 1g, **CP21** dose-dependently promoted the death of HepG2 cells with effects similar to that of BMS-1233 (supplementary Fig. S4a, b). The immunomodulatory effects of **CP21** were further confirmed by the MDB-MB-231& PBMC, Hep3B&PMBC, and Jurkat T&B16-F10 co-culture models (supplementary Fig. S4c–g). Collectively, these results suggest that **CP21** could target both CXCL12 and PD-L1 in vitro.

The pharmacokinetic properties of **CP21** were evaluated in male SD rats with **CP21** administered intravenously (i.v.) and orally (p.o.). As detailed in Table S2, oral gavage of **CP21** (18 mg/kg) exhibited low but acceptable plasma exposure (AUC_(0–t)_= 127.5 ± 4.8 ng/mL h). While intravenous administration of **CP21** (1 mg/kg) displayed more favorable pharmacokinetic properties, such as higher exposure (AUC = 2032.5 ± 11.44.8 ng/mL h), and lower clearance rate (0.5 ± 0.1 L/h/kg).

Having identified the excellent PD-L1/CXCL12 inhibitory potency in vitro for the bifunctional molecule **CP21**, we next evaluated the anticancer activity of **CP21** in vivo using a mouse B16-F10 model. As presented in Fig. 1h, i, **CP21** dose-dependently decreased tumor weights and volumes. Oral administration of **CP21** at 125 mg/kg exhibited a tumor growth inhibition (TGI) of 58.5%, better than either of the monotherapies. In addition, the body weights of mice did not change markedly during the treatment (supplementary Fig. S5). In addition, **CP21** exhibited similarly high in vivo antitumor efficacy in a CT-26 colon syngeneic tumor model (supplementary Fig. S6).

To examine the immunomodulatory effects of **CP21**, a flow cytometry assay was carried out to analyze the TILs (tumor-infiltrating lymphocytes). As shown in supplementary Fig. S7 (representative examples), the percentage of TILs (CD3^+^) in the **CP21** treatment group was dose-dependently higher than that in either of the monotherapy groups. In addition, data from multiple repeated experiments/animals also showed that the percentages of CD3^+^, CD3^+^CD8^+^, and the ratio of CD8^+^/CD4^+^ T cells were dose-dependently/significantly increased in the **CP21** treatment groups, higher than either of the monotherapy groups (Fig. 1j–l). The above results indicate that **CP21** could activate the immune microenvironments in tumors.

In addition, serum biochemistry showed that **CP21** did not induce hepatotoxicity and nephrotoxicity in mice (Supplementary Fig. S8a). Importantly, **CP21** treatment did not cause cardiotoxicity based on the creatine kinase isoenzyme assay (Supplementary Fig. S8b). Furthermore, H&E staining revealed that there was no apparent morphological aberration in the **CP21** treatment group (Supplementary Fig. S8c), which was in agreement with the results of serum biochemistry.

Collectively, **CP21** is a bifunctional small molecule targeting PD-L1 and CXCL12 with high affinities. It also demonstrated high in vivo antitumor efficacy by activating the immune system. Hence, **CP21** represents a first-in-class dual PD-L1/CXCL12 inhibitor deserving further investigation as a potential dual immunotherapy agent for cancer treatment.

## Supplementary information

Supplemental material

## Data Availability

All the data used for the current study are available from the corresponding author upon reasonable request.
