# Supplementary material for: Bifunctional small molecules targeting PD-L1/CXCL12 as dual immunotherapy for cancer treatment
Source: Signal Transduct Target Ther. 2023 Mar 1;8:91. doi: 10.1038/s41392-022-01292-5 (PMC9974934; doi:10.1038/s41392-022-01292-5)
Supplement: Supplementary file 1 — Supplemental material [file 41392_2022_1292_MOESM1_ESM.docx]

Supplementary Materials for

Bifunctional Small Molecules Targeting PD-L1/CXCL12 as Dual Immunotherapy for Cancer Treatment

*Binbin Cheng^1,2#^, Wei Wang^2#^, Ting Liu^2^, Hao Cao^2^, Wei Pan^3^, Yao Xiao^2^, Shuwen Liu^2*^, Jianjun Chen^2*^*

^1^School of Medicine, Hubei Polytechnic University, Hubei Key Laboratory for Kidney Disease Pathogenesis and Intervention, Huangshi 435003, China; ^2^School of Pharmaceutical Sciences, Guangdong Provincial Key Laboratory of New Drug Screening, Southern Medical University, Guangzhou 510515, China; ^3^Department of Cardiology, The Sixth Affiliated Hospital, South China University of Technology, Nanhai people’s Hospital, Foshan, Guangdong 528200, China.

Correspondence to: liusw@smu.edu.cn; [jchen21@smu.edu.cn](mailto:jchen21@smu.edu.cn).

**This PDF file includes:**

Materials and Methods

Figures. S1 to S8

Tables S1 to S2

**Materials and Methods**

General methods

Reagents and anhydrous solvents were commercially available from companies like Sigma-Aldrich. The positive control compounds (BMS-1233) were provided by InvivoChem company (IL, USA, Libertyville). Reactions were detected by GF/UV 254 silica gel and final compounds were purified by Biotage flash column chromatography. Target compounds were identified by NMR (^1^H and ^13^C) acquired from a 400 MHz Bruker instrument (Germany, AV-400 spectrometer). HPLC analysis showed that the purity of all final compounds was ≥95% (Shimadzu LC-20A model, wavelength of 254 nm). High-resolution mass spectrometry (HRMS) was obtained on a Micromass Q-TOF mass spectrometer.

(*E*)-1-(3-bromo-4-((2-methyl-[1,1'-biphenyl]-3-yl)methoxy)benzyl)-*N*-(5-((4-((2-methoxy-4-(3-(4-methoxyphenyl)-3-oxoprop-1-en-1-yl)phenoxy)methyl)benzyl)amino)-5-oxopentyl)piperidine-2-carboxamide (CP1). Yellow oil; ^1^H NMR (*d*6-DMSO) δ 8.27 (t, 1H, *J* = 5.8 Hz), 8.17 (d, 2H, *J* = 8.7 Hz), 7.84 (dd, 2H, *J* = 16.2, 0.5 Hz,), 7.66 (d, 1H, *J* = 15.5 Hz), 7.60 (s, 1H), 7.53 (d, 2H, *J* = 8.7 Hz), 7.45 (t, 2H, *J* = 7.3 Hz), 7.40 – 7.36 (m, 3H), 7.31 (d, 4H, *J* = 7.1 Hz), 7.22 (dd, 4H, *J* = 15.7, 7.2 Hz), 7.09 (dd, 3H, *J* = 8.5, 5.0 Hz), 5.21 (s, 2H), 5.12 (s, 2H), 4.25 (d, 2H, *J* = 5.7 Hz), 3.87 (d, 6H, *J* = 3.6 Hz), 3.65 (d, 1H, *J* = 13.5 Hz), 3.17 (d, 1H, *J* = 4.9 Hz), 3.12 (d, 1H, *J* = 7.4 Hz), 3.08 (d, 1H, *J* = 7.3 Hz), 3.03 (s, 1H), 2.68 (d, 2H, *J* = 7.7 Hz), 2.22 (s, 3H), 2.13 (d, 2H, *J* = 7.1 Hz), 1.99 (d, 1H, *J* = 7.3 Hz), 1.89 (d, 1H, *J* = 9.9 Hz), 1.69 (dd, 3H, *J* = 23.1, 13.2 Hz), 1.53 (dd, 3H, *J* = 10.7, 8.1 Hz), 1.46 – 1.41 (m, 2H). HPLC: *t*_R_ 21.875 min, purity 100%. HRMS (ESI-Q-TOF) *m/z*: [M+H]^+^ calculated for C_57_H_61_BrN_3_O_7_ : 978.3693, found : 978.3677.

(*E*)-1-(3-bromo-4-((2-methyl-[1,1'-biphenyl]-3-yl)methoxy)benzyl)-*N*-(4-((2-methoxy-4-(3-(4-methoxyphenyl)-3-oxoprop-1-en-1-yl)phenoxy)methyl)benzyl)piperidine-2-carboxamide (CP2). Light yellow solid; mp: 124.8–125.9 ºC; ^1^H NMR (*d*6-DMSO) δ 8.42 (t, 1H, *J* = 6.1 Hz), 8.17 (d, 2H, *J* = 8.9 Hz), 7.82 (d, 1H, *J* = 15.5 Hz), 7.65 (d, 1H, *J* = 15.4 Hz), 7.58 (d, 1H, *J* = 1.6 Hz), 7.54 – 7.50 (m, 2H), 7.46 (t, 2H, *J* = 7.3 Hz), 7.38 (s, 2H), 7.31 (dd, 7H, *J* = 14.1, 7.1 Hz), 7.27 – 7.12 (m, 3H), 7.08 (dd, 3H, *J* = 11.2, 8.7 Hz), 5.22 (s, 2H), 5.10 (s, 2H), 4.33 (dd, 2H, *J* = 21.8, 5.9 Hz), 4.14 – 4.07 (m, 2H), 3.86 (d, 6H, *J* = 9.0 Hz), 3.65 (s, 1H), 3.06 (d, 1H, *J* = 13.2 Hz), 2.77 (d, 2H, *J* = 12.2 Hz), 2.22 (s, 3H), 1.89 (d, 1H, *J* = 13.6 Hz), 1.77 (d, 1H, *J* = 14.1 Hz), 1.70 – 1.65 (m, 1H), 1.62 (d, 1H, *J* = 12.4 Hz), 1.53 (s, 1H), 1.38 (d, 1H, *J* = 8.4 Hz). ^13^C NMR (*d*6-DMSO) δ 187.72, 173.60, 163.48, 153.82, 150.48, 149.69, 143.98, 142.52, 141.69, 140.02, 135.70, 135.53, 134.15, 133.58, 132.75, 131.22, 131.08, 129.99, 129.83, 129.57, 128.65, 128.28, 127.91, 127.70, 127.37, 125.92, 123.91, 120.11, 114.36, 113.96, 113.55, 111.47, 111.33, 70.09, 69.66, 67.53, 58.65, 56.20, 55.96, 51.22, 49.00, 42.11, 30.04, 26.97, 24.97, 23.53, 21.16, 16.21. HPLC: *t*_R_ 22.734 min, purity 100%. HRMS (ESI-Q-TOF) *m/z*: [M+H]^+^ calculated for C_52_H_52_BrN_2_O_6_ : 879.3009, found: 879.2994.

(*E*)-1-(1-(3-bromo-4-((2-methyl-[1,1'-biphenyl]-3-yl)methoxy)benzyl)piperidine-2-carbonyl)-*N*-(4-((2-methoxy-4-(3-(4-methoxyphenyl)-3-oxoprop-1-en-1-yl)phenoxy)methyl)benzyl)piperidine-4-carboxamide (CP3). Yellow oil; ^1^H NMR (*d*6-DMSO) δ 8.37 (d, 1H, *J* = 5.9 Hz), 8.17 (d, 2H, *J* = 8.8 Hz), 7.83 (d, 1H, *J* = 15.5 Hz), 7.66 (d, 1H, *J* = 15.5 Hz), 7.53 (d, 3H, *J* = 7.8 Hz), 7.46 (t, 2H, *J* = 7.4 Hz), 7.40 (d, 2H, *J* = 7.8 Hz), 7.35 (d, 1H, *J* = 10.2 Hz), 7.31 (d, 3H, *J* = 7.2 Hz), 7.23 (dd, 5H, *J* = 19.1, 6.6 Hz), 7.09 (d, 3H, *J* = 8.7 Hz), 5.23 (s, 2H), 5.13 (s, 2H), 4.43 (d, 2H, *J* = 15.4 Hz), 4.27 (d, 2H, *J* = 3.4 Hz), 3.87 (d, 6H, *J* = 2.9 Hz), 3.71 – 3.54 (m, 2H), 3.17 (d, 2H, *J* = 3.1 Hz), 3.12 (d, 1H, *J* = 7.5 Hz), 3.04 (d, 2H, *J* = 11.7 Hz), 2.84 (d, 1H, *J* = 4.7 Hz), 2.69 – 2.56 (m, 2H), 1.99 (d, 1H, *J* = 2.4 Hz), 1.76 (d, 2H, *J* = 13.0 Hz), 1.64 (d, 2H, *J* = 5.3 Hz), 1.51 – 1.34 (m, 5H). HPLC: *t*_R_ 21.682 min, purity 95.907%. HRMS (ESI-Q-TOF) *m/z*: [M+H]^+^ calculated for C_58_H_61_BrN_3_O_7_: 990.3693, found: 990.3660.

(*E*)-1-(3-bromo-4-((2-methyl-[1,1'-biphenyl]-3-yl)methoxy)benzyl)-*N*-(4-((4-((2-methoxy-4-(3-(4-methoxyphenyl)-3-oxoprop-1-en-1-yl)phenoxy)methyl)benzyl)amino)-4-oxobutyl)piperidine-2-carboxamide (CP4). Yellow oil; ^1^H NMR (*d*6-DMSO) δ 8.32 (s, 1H), 8.17 (d, 2H, *J* = 8.9 Hz), 7.92 (s, 1H), 7.82 (d, 1H, *J* = 15.4 Hz), 7.72 – 7.59 (m, 3H), 7.57 – 7.43 (m, 5H), 7.39 (d, 3H, *J* = 8.0 Hz), 7.32 (s, 3H), 7.28 – 7.19 (m, 4H), 7.12 – 7.06 (m, 2H), 5.22 (s, 2H), 5.12 (s, 2H), 4.26 (d, 2H, *J* = 6.0 Hz), 3.86 (d, 6H, *J* = 10.5 Hz), 3.68 – 3.61 (m, 2H), 3.18 (d, 2H, *J* = 5.8 Hz), 3.12 (s, 2H), 2.75 (d, 2H, *J* = 4.2 Hz), 2.68 (d, 2H, *J* = 6.0 Hz), 2.22 (s, 3H), 2.17 – 2.12 (m, 2H), 1.99 (d, 2H, *J* = 5.3 Hz), 1.67 (d, 2H, *J* = 7.7 Hz), 1.48 (d, 2H, *J* = 13.4 Hz). HPLC: *t*_R_ 21.662 min, purity 100%. HRMS (ESI-Q-TOF) *m/z*: [M+H]^+^ calculated for C_56_H_59_BrN_3_O_7_: 964.3536, found: 964.3525.

(*E*)-1-(3-bromo-4-((2-methyl-[1,1'-biphenyl]-3-yl)methoxy)benzyl)-*N*-(3-((4-((2-methoxy-4-(3-(4-methoxyphenyl)-3-oxoprop-1-en-1-yl)phenoxy)methyl)benzyl)amino)-3-oxopropyl)piperidine-2-carboxamide (CP5). Yellow oil; ^1^H NMR (*d*6-DMSO) δ 8.38 (t, 1H, *J* = 5.9 Hz), 8.16 (d, 2H, *J* = 8.7 Hz), 7.89 (s, 1H), 7.82 (d, 1H, *J* = 15.5 Hz), 7.66 (d, 1H, *J* = 15.4 Hz), 7.59 (s, 1H), 7.52 (d, 2H, *J* = 10.5 Hz), 7.46 (t, 2H, *J* = 7.2 Hz), 7.36 (d, 3H, *J* = 8.2 Hz), 7.31 (dd, 4H, *J* = 12.3, 5.5 Hz), 7.24 – 7.20 (m, 3H), 7.14 – 7.03 (m, 3H), 5.21 (s, 2H), 5.12 (s, 2H), 4.22 (dt, 2H, *J* = 10.6, 6.9 Hz), 3.87 (d, 6H, *J* = 5.9 Hz), 3.67 (s, 1H), 3.01 (d, 1H, *J* = 13.6 Hz), 2.73 – 2.66 (m, 2H), 2.37 (t, 2H, *J* = 6.6 Hz), 2.22 (s, 3H), 2.04 – 1.98 (m, 1H), 1.85 (t, 1H, *J* = 9.7 Hz), 1.70 (s, 1H), 1.64 (d, 1H, *J* = 9.0 Hz), 1.51 (d, 3H, *J* = 10.1 Hz), 1.33 (d, 2H, *J* = 10.1 Hz). HPLC: *t*_R_ 21.997 min, purity 97.381%. HRMS (ESI-Q-TOF) *m/z*: [M+H]^+^ calculated for C_55_H_57_BrN_3_O_7_: 950.3380, found: 950.3367.

(*E*)-1-(5-chloro-4-((2-methyl-[1,1'-biphenyl]-3-yl)methoxy)-2-((4-(trifluoromethoxy)benzyl)oxy)benzyl)-*N*-(4-((2-methoxy-4-(3-(4-methoxyphenyl)-3-oxoprop-1-en-1-yl)phenoxy)methyl)benzyl)piperidine-2-carboxamide (CP6). Yellow oil; ^1^H NMR (*d*6-DMSO) δ 8.28 (t, 1H, *J* = 6.4 Hz), 8.16 (d, 2H, *J* = 8.7 Hz), 7.82 (d, 1H, *J* = 15.5 Hz), 7.65 (d, 1H, *J* = 15.4 Hz), 7.60 – 7.55 (m, 2H), 7.53 (s, 1H), 7.47 (dd, 4H, *J* = 15.2, 7.2 Hz), 7.39 (d, 3H, *J* = 8.5 Hz), 7.31 (d, 5H, *J* = 7.3 Hz), 7.28 (d, 1H, *J* = 7.6 Hz), 7.21 (d, 3H, *J* = 7.8 Hz), 7.08 (dd, 3H, *J* = 13.3, 10.3 Hz), 5.23 (d, 4H, *J* = 9.2 Hz), 5.08 (s, 2H), 4.25 (dt, 2H, *J* = 15.1, 9.0 Hz), 3.86 (d, 6H, *J* = 11.4 Hz), 3.48 (s, 1H), 2.78 (d, 2H, *J* = 15.6 Hz), 2.24 (s, 3H), 2.04 – 1.97 (m, 1H), 1.93 (t, 1H, *J* = 12.6 Hz), 1.80 – 1.74 (m, 1H), 1.72 – 1.56 (m, 3H), 1.54 – 1.49 (m, 1H), 1.44 (d, 1H, *J* = 4.1 Hz). ^13^C NMR (*d*6-DMSO) δ 187.70, 173.55, 163.75, 156.12, 153.45, 150.48, 149.76, 143.91, 142.64, 141.74, 139.91, 136.84, 135.54, 134.38, 131.16, 129.80, 129.56, 128.66, 128.26, 127.47, 125.96, 123.90, 121.49, 120.66, 120.12, 114.35, 113.51, 111.48, 100.91, 70.07, 69.51, 67.81, 56.18, 55.96, 55.33, 53.44, 31.68, 30.83, 30.12, 16.22. HPLC: *t*_R_ 24.243 min, purity 99.488%. HRMS (ESI-Q-TOF) *m/z*: [M+H]^+^ calculated for C_60_H_57_ClF_3_N_2_O_8_: 1025.3756, found: 1025.3754.

(*E*)-1-(1-(5-chloro-4-((2-methyl-[1,1'-biphenyl]-3-yl)methoxy)-2-((4-(trifluoromethoxy)benzyl)oxy)benzyl)piperidine-2-carbonyl)-*N*-(4-((2-methoxy-4-(3-(4-methoxyphenyl)-3-oxoprop-1-en-1-yl)phenoxy)methyl)benzyl)piperidine-4-carboxamide (CP7). Yellow oil; ^1^H NMR (*d*6-DMSO) δ 8.35 (s, 1H), 8.17 (d, 2H, *J* = 8.8 Hz), 7.82 (d, 1H, *J* = 15.5 Hz), 7.66 (d, 1H, *J* = 15.5 Hz), 7.60 (d, 2H, *J* = 8.5 Hz), 7.50 (dd, 3H, *J* = 19.2, 11.2 Hz), 7.37 (ddd, 7H, *J* = 22.4, 16.2, 8.9 Hz), 7.23 (t, 3H, *J* = 13.4 Hz), 7.13 – 7.02 (m, 3H), 5.31 – 5.05 (m, 6H), 4.38 (d, 2H, *J* = 10.9 Hz), 4.26 (d, 2H, *J* = 2.6 Hz), 3.87 (d, 4H, *J* = 9.7 Hz), 3.54 – 3.45 (m, 2H), 3.06 – 2.84 (m, 5H), 2.68 (s, 2H), 2.23 (s, 3H), 2.01 (dd, 4H, *J* = 15.3, 7.1 Hz), 1.68 (dd, 5H, *J* = 30.0, 6.4 Hz), 1.48 – 1.28 (m, 6H). HPLC: *t*_R_ 22.679 min, purity 99.012%. HRMS (ESI-Q-TOF) *m/z*: [M+H]^+^ calculated for C_66_H_66_ClF_3_N_3_O_9_: 1136.4440, found: 1136.4421.

(*E*)-1-(5-chloro-4-((2-methyl-[1,1'-biphenyl]-3-yl)methoxy)-2-((4-(trifluoromethoxy)benzyl)oxy)benzyl)-*N*-(4-((4-((2-methoxy-4-(3-(4-methoxyphenyl)-3-oxoprop-1-en-1-yl)phenoxy)methyl)benzyl)amino)-4-oxobutyl)piperidine-2-carboxamide (CP8). Yellow oil; ^1^H NMR (*d*6-DMSO) δ 8.29 (d, 1H, *J* = 6.3 Hz), 8.17 (d, 2H, *J* = 8.8 Hz), 7.80 (t, 2H, *J* = 12.4 Hz), 7.68 (s, 1H), 7.60 (d, 2H, *J* = 8.5 Hz), 7.54 (s, 1H), 7.47 (dd, 4H, *J* = 13.6, 7.4 Hz), 7.43 – 7.36 (m, 5H), 7.35 – 7.28 (m, 4H), 7.27 – 7.21 (m, 3H), 7.08 (t, 3H, *J* = 7.5 Hz), 5.24 (d, 4H, *J* = 5.4 Hz), 5.11 (s, 2H), 4.25 (d, 2H, *J* = 5.6 Hz), 3.87 (d, 6H, *J* = 5.5 Hz), 2.73 (dd, 3H, *J* = 10.0, 6.4 Hz), 2.23 (s, 3H), 2.11 (t, 2H, *J* = 7.3 Hz), 2.02 – 1.98 (m, 1H), 1.94 – 1.83 (m, 2H), 1.75 (s, 1H), 1.68 – 1.61 (m, 3H), 1.51 (dd, 3H, *J* = 22.5, 8.2 Hz), 1.37 (dd, 2H, *J* = 11.9, 4.8 Hz). HPLC: *t*_R_ 23.384 min, purity 97.701%. HRMS (ESI-Q-TOF) *m/z*: [M+H]^+^ calculated for C_64_H_64_ClF_3_N_3_O_9_: 1110.4283, found: 1110.4293.

(*E*)-4-(2-((5-chloro-2-((3-cyanobenzyl)oxy)-4-((2-methyl-[1,1'-biphenyl]-3-yl)methoxy)benzyl)amino)-2-methylpropanamido)-*N*-(4-((2-methoxy-4-(3-(4-methoxyphenyl)-3-oxoprop-1-en-1-yl)phenoxy)methyl)benzyl)butanamide (CP9). Yellow oil; ^1^H NMR (*d*6-DMSO) δ 8.31 (d, 1H, *J* = 5.8 Hz), 8.17 (d, 2H, *J* = 8.6 Hz), 7.95 (s, 1H), 7.86 – 7.73 (m, 4H), 7.71 – 7.50 (m, 4H), 7.46 (dd, 4H, *J* = 13.2, 5.8 Hz), 7.37 (t, 3H, *J* = 7.6 Hz), 7.31 (d, 3H, *J* = 7.8 Hz), 7.27 (s, 2H), 7.21 (s, 1H), 7.08 (dd, 3H), 5.30 (s, 2H, *J* = 10.2, 4.9 Hz), 5.23 (s, 2H), 5.12 (s, 2H), 4.25 (d, 2H, *J* = 5.6 Hz), 3.87 (d, 6H, *J* = 5.0 Hz), 3.50 (s, 2H), 3.03 (d, 2H, *J* = 6.1 Hz), 2.23 (s, 3H), 2.12 (t, 2H, *J* = 7.4 Hz), 2.04 – 1.95 (m, 2H), 1.66 – 1.60 (m, 2H), 1.20 (s, 6H). HPLC: *t*_R_ 20.848 min, purity 95.976%. HRMS (ESI-Q-TOF) *m/z*: [M+H]^+^ calculated for C_62_H_62_ClN_4_O_8_: 1025.4256, found: 1025.4243.

(*E*)-2-((5-chloro-2-((3-cyanobenzyl)oxy)-4-((2-methyl-[1,1'-biphenyl]-3-yl)methoxy)benzyl)amino)-*N*-(4-((2-methoxy-4-(3-(4-methoxyphenyl)-3-oxoprop-1-en-1-yl)phenoxy)methyl)benzyl)-2-methylpropanamide (CP10). Yellow oil; ^1^H NMR (*d*6-DMSO) δ 8.29 (t, 1H, *J* = 6.2 Hz), 8.17 (d, 2H, *J* = 8.7 Hz), 7.99 – 7.89 (m, 2H), 7.85 – 7.74 (m, 3H), 7.65 (d, 1H, *J* = 15.5 Hz), 7.60 – 7.52 (m, 2H), 7.46 (dd, 3H, *J* = 12.9, 5.6 Hz), 7.39 (d, 2H, *J* = 9.0 Hz), 7.36 – 7.31 (m, 3H), 7.30 – 7.26 (m, 2H), 7.22 (dd, 3H, *J* = 12.7, 7.8 Hz), 7.08 (t, 2H, *J* = 8.2 Hz), 7.04 (s, 1H), 6.91 (dd, 1H, *J* = 17.3, 10.8 Hz), 5.24 (d, 4H, *J* = 13.1 Hz), 5.10 (s, 2H), 4.41 – 4.30 (m, 1H), 4.27 (d, 2H, *J* = 6.1 Hz), 3.87 (d, 6H, *J* = 6.5 Hz), 3.61 (s, 1H), 3.52 (s, 2H), 2.22 (s, 3H), 1.25 (s, 6H). HPLC: *t*_R_ 21.515 min, purity 98.528%. HRMS (ESI-Q-TOF) *m/z*: [M+H]^+^ calculated for C_58_H_55_ClN_3_O_7_: 940.3729, found: 940.3713.

(*E*)-2-((5-chloro-2-((3-cyanobenzyl)oxy)-4-((2-methyl-[1,1'-biphenyl]-3-yl)methoxy)benzyl)amino)-*N*-(3-((4-((2-methoxy-4-(3-(4-methoxyphenyl)-3-oxoprop-1-en-1-yl)phenoxy)methyl)benzyl)amino)-3-oxopropyl)-2-methylpropanamide (CP11). Yellow oil; ^1^H NMR (*d*6-DMSO) δ 8.37 (s, 1H), 8.16 (d, 2H, *J* = 8.8 Hz), 7.94 (d, 2H, *J* = 12.8 Hz), 7.89 – 7.75 (m, 3H), 7.72 – 7.49 (m, 4H), 7.46 (s, 3H), 7.39 (d, 1H, *J* = 7.5 Hz), 7.36 (d, 2H, *J* = 7.9 Hz), 7.31 (d, 2H, *J* = 8.2 Hz), 7.27 (s, 1H), 7.24 – 7.19 (m, 2H), 7.18 – 6.93 (m, 4H), 5.27 (d, 4H, *J* = 29.4 Hz), 5.11 (s, 2H), 4.23 (d, 2H, *J* = 5.6 Hz), 3.87 (d, 6H, *J* = 6.7 Hz), 3.47 (d, 2H, *J* = 5.2 Hz), 3.30 (s, 2H), 2.36 – 2.28 (m, 2H), 2.22 (s, 3H), 2.00 (t, 2H, *J* = 10.1 Hz), 1.51 – 1.41 (m, 1H), 1.18 (s, 6H). HPLC: *t*_R_ 20.879 min, purity 95.343%. HRMS (ESI-Q-TOF) *m/z*: [M+H]^+^ calculated for C_61_H_60_ClN_4_O_8_: 1011.4100, found:1011.4099.

(*E*)-5-(2-((5-chloro-2-((3-cyanobenzyl)oxy)-4-((2-methyl-[1,1'-biphenyl]-3-yl)methoxy)benzyl)amino)-2-methylpropanamido)-*N*-(4-((2-methoxy-4-(3-(4-methoxyphenyl)-3-oxoprop-1-en-1-yl)phenoxy)methyl)benzyl)pentanamide (CP12). Yellow oil; ^1^H NMR (*d*6-DMSO) δ 8.26 (d, 1H, *J* = 5.7 Hz), 8.16 (d, 2H, *J* = 8.8 Hz), 7.95 (s, 2H), 7.82 (d, 3H, *J* = 15.2 Hz), 7.74 (s, 1H), 7.63 (dd, 2H, *J* = 20.2, 12.1 Hz), 7.54 (s, 1H), 7.46 (dd, 4H, *J* = 14.9, 7.5 Hz), 7.38 (d, 3H, *J* = 7.6 Hz), 7.31 (d, 2H, *J* = 7.1 Hz), 7.28 – 7.18 (m, 4H), 7.10 – 7.06 (m, 3H), 5.30 (s, 2H), 5.23 (s, 2H), 5.11 (s, 2H), 4.25 (d, 2H, *J* = 5.8 Hz), 3.87 (d, 6H, *J* = 4.9 Hz), 3.49 (s, 2H), 3.01 (d, 2H, *J* = 6.3 Hz), 2.23 (s, 3H), 2.11 (t, 2H, *J* = 7.2 Hz), 2.02 – 1.93 (m, 2H), 1.49 – 1.43 (m, 2H), 1.38 – 1.33 (m, 2H), 1.19 (s, 6H). HPLC: *t*_R_ 20.946 min, purity 98.747%. HRMS (ESI-Q-TOF) *m/z*: [M+H]^+^ calculated for C_63_H_64_ClN_4_O_8_: 1039.4413, found:1039.4401.

ethyl (*E*)-2-(4-chloro-2-((2-((3-((4-((2-methoxy-4-(3-(4-methoxyphenyl)-3-oxoprop-1-en-1-yl)phenoxy)methyl)benzyl)amino)-3-oxopropyl)carbamoyl)piperidin-1-yl)methyl)-5-((2-methyl-[1,1'-biphenyl]-3-yl)methoxy)phenoxy)acetate (CP13). Yellow oil; ^1^H NMR (*d*6-DMSO) δ 8.36 (s, 1H), 8.16 (d, 2H, *J* = 8.7 Hz), 7.82 (d, 2H, *J* = 15.4 Hz), 7.67 (s, 1H), 7.54 (s, 1H), 7.50 (s, 1H), 7.48 (s, 1H), 7.46 (d, 3H, *J* = 4.3 Hz), 7.38 (s, 1H), 7.37 (s, 1H), 7.35 (s, 1H), 7.32 (s, 2H), 7.31 (s, 1H), 7.28 (d, 1H, *J* = 7.1 Hz), 7.23 (s, 1H), 7.21 (s, 1H), 7.11 – 7.06 (m, 3H), 6.94 (s, 1H), 5.23 (s, 2H), 5.11 (s, 2H), 4.91 (s, 2H), 4.24 (d, 2H, *J* = 5.8 Hz), 4.15 (s, 2H), 3.87 (d, 6H, *J* = 6.2 Hz), 2.70 (s, 1H), 2.34 (s, 3H), 2.22 (s, 3H), 1.89 (d, *J* = 6.7 Hz, 2H), 1.75 – 1.71 (m, 2H), 1.63 (d, 2H, *J* = 9.7 Hz), 1.50 – 1.46 (m, 3H), 1.20 (s, 1H), 1.19 (s, 1H), 1.17 (s, 1H). HPLC: *t*_R_ 21.563 min, purity 95.959%. HRMS (ESI-Q-TOF) *m/z*: [M+H]^+^ calculated for C_59_H_63_ClN_3_O_10_: 1008.4202, found:1008.4204.

ethyl (*E*)-2-(4-chloro-2-((2-((4-((2-methoxy-4-(3-(4-methoxyphenyl)-3-oxoprop-1-en-1-yl)phenoxy)methyl)benzyl)carbamoyl)piperidin-1-yl)methyl)-5-((2-methyl-[1,1'-biphenyl]-3-yl)methoxy)phenoxy)acetate (CP14). Yellow oil; ^1^H NMR (*d*6-DMSO) δ 8.33 (t, 1H, *J* = 6.0 Hz), 8.17 (d, 2H, *J* = 8.8 Hz), 7.82 (d, 1H, *J* = 15.5 Hz), 7.65 (d, 1H, *J* = 15.4 Hz), 7.54 – 7.43 (m, 5H), 7.39 (d, 1H, *J* = 7.4 Hz), 7.32 (d, 5H, *J* = 10.0 Hz), 7.28 – 7.17 (m, 4H), 7.12 – 7.03 (m, 3H), 6.93 (s, 1H), 5.23 (s, 2H), 5.10 (s, 2H), 4.86 (s, 2H), 4.31 (dt, 2H, *J* = 15.0, 9.2 Hz), 4.16 – 4.10 (m, 2H), 3.87 (d, 6H, *J* = 7.9 Hz), 3.55 (d, 1H, *J* = 13.9 Hz), 3.18 (d, 1H, *J* = 5.1 Hz), 2.81 (d, 2H, *J* = 9.3 Hz), 2.23 (s, 3H), 1.94 (s, 1H), 1.79 (d, 1H, *J* = 11.6 Hz), 1.67 (dd, 2H, *J* = 13.4, 4.2 Hz), 1.51 (s, 1H), 1.42 – 1.37 (m, 1H), 1.31 (s, 1H), 1.18 (t, 3H, *J* = 7.1 Hz). HPLC: *t*_R_ 22.296 min, purity 95.312%. HRMS (ESI-Q-TOF) *m/z*: [M+H]^+^ calculated for C_56_H_58_ClN_2_O_9_: 937.3831, found:937.3831.

ethyl (*E*)-2-(4-chloro-2-((2-(4-((4-((2-methoxy-4-(3-(4-methoxyphenyl)-3-oxoprop-1-en-1-yl)phenoxy)methyl)benzyl)carbamoyl)piperidine-1-carbonyl)piperidin-1-yl)methyl)-5-((2-methyl-[1,1'-biphenyl]-3-yl)methoxy)phenoxy)acetate (CP15). Yellow oil; ^1^H NMR (*d*6-DMSO) δ 8.36 (s, 1H), 8.17 (d, 2H, *J* = 8.9 Hz), 7.83 (d, 1H, *J* = 15.5 Hz), 7.66 (d, 1H, *J* = 15.4 Hz), 7.55 – 7.44 (m, 4H), 7.42 – 7.28 (m, 8H), 7.24 (dd, 4H, *J* = 21.6, 8.7 Hz), 7.09 (d, 3H, *J* = 8.8 Hz), 6.94 (s, 1H), 5.23 (s, 2H), 5.13 (s, 2H), 4.89 (s, 2H), 4.40 (d, 1H, *J* = 12.7 Hz), 4.26 (s, 2H), 4.15 (dd, 2H, *J* = 14.0, 6.7 Hz), 3.87 (d, 6H, *J* = 2.8 Hz), 3.56 (dd, 2H, *J* = 40.1, 36.0 Hz), 3.03 (d, 2H, *J* = 12.0 Hz), 2.92 (d, 2H, *J* = 12.4 Hz), 2.59 (d, 1H, *J* = 5.4 Hz), 2.44 (s, 2H), 2.23 (s, 3H), 2.05 – 1.96 (m, 2H), 1.76 (s, 2H), 1.67 (d, 3H, *J* = 10.1 Hz), 1.46 (d, 2H, *J* = 3.7 Hz), 1.19 (d, 3H, *J* = 7.1 Hz). HPLC: *t*_R_ 21.009 min, purity 99.412%. HRMS (ESI-Q-TOF) *m/z*: [M+H]^+^ calculated for C_62_H_67_ClN_3_O_10_: 1048.4515, found: 1048.4525.

ethyl (*E*)-2-(4-chloro-2-((2-((4-((4-((2-methoxy-4-(3-(4-methoxyphenyl)-3-oxoprop-1-en-1-yl)phenoxy)methyl)benzyl)amino)-4-oxobutyl)carbamoyl)piperidin-1-yl)methyl)-5-((2-methyl-[1,1'-biphenyl]-3-yl)methoxy)phenoxy)acetate (CP16). Yellow oil; ^1^H NMR (*d*6-DMSO) δ 8.30 (t, 1H, *J* = 6.1 Hz), 8.17 (d, 2H, *J* = 8.8 Hz), 7.82 (d, 2H, *J* = 15.8 Hz), 7.66 (d, 1H, *J* = 15.4 Hz), 7.55 – 7.44 (m, 5H), 7.39 (d, 3H, *J* = 8.1 Hz), 7.33 – 7.29 (m, 3H), 7.26 (d, 2H, *J* = 8.1 Hz), 7.21 (d, 1H, *J* = 7.3 Hz), 7.09 (dd, 3H, *J* = 8.4, 4.6 Hz), 6.94 (s, 1H), 5.23 (s, 2H), 5.12 (s, 2H), 4.90 (s, 2H), 4.26 (d, 2H, *J* = 5.6 Hz), 4.15 (q, 2H, *J* = 7.1 Hz), 3.87 (d, 6H, *J* = 4.4 Hz), 3.10 (dd, 3H, *J* = 11.3, 5.0 Hz), 2.79 (d, 2H, *J* = 13.6 Hz), 2.71 (d, 2H, *J* = 13.0 Hz), 2.23 (s, 3H), 2.12 (d, 2H, *J* = 7.1 Hz), 2.02 – 1.89 (m, 3H), 1.75 (d, 1H, *J* = 11.5 Hz), 1.68 – 1.64 (m, 2H), 1.52 (dd, 2H, *J* = 24.4, 12.1 Hz), 1.37 (d, 1H, *J* = 9.0 Hz), 1.19 (t, 3H, *J* = 7.1 Hz). HPLC: *t*_R_ 21.776 min, purity 95.685%. HRMS (ESI-Q-TOF) *m/z*: [M+H]^+^ calculated for C_60_H_65_ClN_3_O_10_: 1022.4358, found: 1022.4350.

(*E*)-1-(5-chloro-2-((3-cyanobenzyl)oxy)-4-((2-methyl-[1,1'-biphenyl]-3-yl)methoxy)benzyl)-*N*-(4-((2-methoxy-4-(3-(4-methoxyphenyl)-3-oxoprop-1-en-1-yl)phenoxy)methyl)benzyl)piperidine-2-carboxamide (CP17). Yellow solid; mp: 128.1–128.9 ºC; ^1^H NMR (*d*6-DMSO) δ 8.27 (s, 1H), 8.17 (d, 2H, *J* = 8.9 Hz), 7.90 (s, 1H), 7.79 (d, 1H, *J* = 4.9 Hz), 7.69 – 7.56 (m, 1H), 7.53 (s, 1H), 7.47 (dd, 4H, *J* = 11.6, 4.5 Hz), 7.39 (d, 4H, *J* = 7.3 Hz), 7.31 (d, 4H, *J* = 7.8 Hz), 7.27 (d, 3H, *J* = 7.5 Hz), 7.21 (d, 3H, *J* = 7.8 Hz), 7.12 – 7.04 (m, 3H), 5.24 (d, 4H, *J* = 7.2 Hz), 5.09 (s, 1H), 4.25 (dd, 2H, *J* = 11.7, 7.8 Hz), 3.87 (d, 6H, *J* = 9.9 Hz), 3.53 (d, 2H, *J* = 12.9 Hz), 2.85 – 2.78 (m, 2H), 2.23 (s, 3H), 2.03 – 1.92 (m, 1H), 1.78 (d, 1H, *J* = 10.5 Hz), 1.66 (dd, 1H, *J* = 17.3, 6.3 Hz), 1.53 (d, 1H, *J* = 17.1 Hz), 1.47 – 1.39 (m, 0H). ^13^C NMR (*d*6-DMSO) δ 187.70, 173.61, 163.49, 155.83, 150.48, 149.73, 143.97, 142.59, 141.69, 139.97, 138.99, 138.91, 138.82, 135.54, 135.50, 134.32, 132.68, 131.34, 131.22, 131.12, 131.10, 131.05, 131.03, 130.22, 130.19, 130.16, 129.57, 128.66, 128.21, 127.55, 127.39, 125.99, 125.97, 123.91, 123.89, 121.42, 120.66, 120.13, 119.85, 119.04, 117.07, 114.47, 114.40, 114.36, 113.64, 113.57, 113.54, 111.90, 111.50, 100.86, 70.03, 70.00, 62.16, 56.25, 55.96, 42.02, 29.48, 28.98, 27.05, 16.32. HPLC: *t*_R_ 22.405 min, purity 97.887%. HRMS (ESI-Q-TOF) *m/z*: [M+H]^+^ calculated for C_60_H_57_ClN_3_O_7_: 966.3885, found: 966.3895.

(*E*)-1-(5-chloro-2-((3-cyanobenzyl)oxy)-4-((2-methyl-[1,1'-biphenyl]-3-yl)methoxy)benzyl)-*N*-(4-((4-((2-methoxy-4-(3-(4-methoxyphenyl)-3-oxoprop-1-en-1-yl)phenoxy)methyl)benzyl)amino)-4-oxobutyl)piperidine-2-carboxamide (CP18). Yellow solid; mp: 120.1–120.9 ºC; ^1^H NMR (*d*6-DMSO) δ 8.29 (t, 1H, *J* = 6.0 Hz), 8.17 (d, 2H, *J* = 8.8 Hz), 7.94 (s, 1H), 7.81 (dd, 4H, *J* = 17.3, 9.0 Hz), 7.63 (dd, 2H, *J* = 19.1, 11.5 Hz), 7.54 (s, 1H), 7.47 (t, 4H, *J* = 8.2 Hz), 7.38 (dd, 3H, *J* = 14.3, 6.4 Hz), 7.34 – 7.28 (m, 3H), 7.25 (d, 2H, *J* = 8.2 Hz), 7.21 (d, 1H, *J* = 7.3 Hz), 7.13 – 7.05 (m, 3H), 5.26 (d, 4H, *J* = 24.1 Hz), 5.12 (s, 2H), 4.25 (d, 2H, *J* = 5.8 Hz), 3.87 (d, 6H, *J* = 5.2 Hz), 3.53 (d, 2H, *J* = 13.7 Hz), 3.29 – 3.21 (m, 2H), 3.02 (dd, 2H, *J* = 17.6, 8.6 Hz), 2.74 (dd, 2H, *J* = 21.6, 8.1 Hz), 2.20 (s, 3H), 2.11 (t, 2H, *J* = 7.2 Hz), 2.03 – 1.96 (m, 1H), 1.91 (t, 1H, *J* = 11.3 Hz), 1.74 (d, 1H, *J* = 12.3 Hz), 1.63 (dd, 2H, *J* = 13.9, 7.0 Hz), 1.52 (d, 2H, *J* = 17.5 Hz), 1.39 (d, 1H, *J* = 12.8 Hz). HPLC: *t*_R_ 21.591 min, purity 100%. HRMS (ESI-Q-TOF) *m/z*: [M+H]^+^ calculated for C_64_H_64_ClN_4_O_8_: 1051.4413, found: 1051.4438.

(*E*)-1-(5-chloro-2-((3-cyanobenzyl)oxy)-4-((2-methyl-[1,1'-biphenyl]-3-yl)methoxy)benzyl)-*N*-(5-((4-((2-methoxy-4-(3-(4-methoxyphenyl)-3-oxoprop-1-en-1-yl)phenoxy)methyl)benzyl)amino)-5-oxopentyl)piperidine-2-carboxamide (CP19). Yellow oil; ^1^H NMR (*d*6-DMSO) δ 8.25 (s, 1H), 8.16 (d, 2H, *J* = 8.7 Hz), 7.82 (d, 1H, *J* = 15.6 Hz), 7.67 (t, 2H, *J* = 13.8 Hz), 7.60 (d, 2H, *J* = 8.5 Hz), 7.54 (s, 1H), 7.47 (dd, 4H, *J* = 13.3, 7.5 Hz), 7.39 (dd, 5H, *J* = 13.8, 8.4 Hz), 7.32 (d, 2H, *J* = 7.2 Hz), 7.28 (d, 1H, *J* = 7.4 Hz), 7.22 (t, 3H, *J* = 8.6 Hz), 7.08 (t, 3H, *J* = 7.5 Hz), 5.24 (d, 4H, *J* = 3.3 Hz), 5.11 (s, 2H), 4.24 (d, 2H, *J* = 5.4 Hz), 3.87 (d, 6H, *J* = 5.0 Hz), 3.60 – 3.47 (m, 2H), 3.06 – 2.89 (m, 4H), 2.73 (dd, 3H, *J* = 22.8, 11.8 Hz), 2.23 (s, 3H), 2.10 (t, 2H, *J* = 7.2 Hz), 2.04 – 1.94 (m, 2H), 1.90 (d, 1H, *J* = 7.9 Hz), 1.71 (d, 1H, *J* = 7.2 Hz), 1.64 (d, 1H, *J* = 6.5 Hz), 1.53 (s, 1H), 1.47 (d, 2H, *J* = 6.4 Hz), 1.37 – 1.32 (m, 2H). HPLC: *t*_R_ 21.753 min, purity 100%. HRMS (ESI-Q-TOF) *m/z*: [M+H]^+^ calculated for C_65_H_66_ClN_4_O_8_: 1065.4569, found: 1065.4575.

(*E*)-1-(5-chloro-2-((3-cyanobenzyl)oxy)-4-((2-methyl-[1,1'-biphenyl]-3-yl)methoxy)benzyl)-*N*-(3-((4-((2-methoxy-4-(3-(4-methoxyphenyl)-3-oxoprop-1-en-1-yl)phenoxy)methyl)benzyl)amino)-3-oxopropyl)piperidine-2-carboxamide (CP20). Yellow oil; ^1^H NMR (*d*6-DMSO) δ 8.35 (s, 1H), 8.16 (d, 2H, *J* = 8.8 Hz), 7.93 (s, 1H), 7.84 – 7.77 (m, 4H), 7.63 (dd, 3H, *J* = 22.7, 11.7 Hz), 7.53 (s, 1H), 7.45 (s, 4H), 7.40 (s, 1H), 7.36 (s, 2H), 7.30 (s, 2H), 7.26 (s, 1H), 7.21 (d, 3H, *J* = 7.6 Hz), 7.10 (s, 2H), 7.05 (s, 2H), 5.31 (s, 2H), 5.22 (s, 2H), 5.11 (s, 2H), 4.23 – 4.16 (m, 2H), 3.87 (d, 6H, *J* = 6.9 Hz), 2.32 (d, 3H, *J* = 6.8 Hz), 2.22 (s, 3H), 2.00 (d, 2H, *J* = 5.1 Hz), 1.91 – 1.85 (m, 2H), 1.73 (d, 2H, *J* = 11.5 Hz), 1.64 (d, 2H, *J* = 6.3 Hz), 1.53 – 1.45 (m, 4H). HPLC: *t*_R_ 21.219 min, purity 97.625%. HRMS (ESI-Q-TOF) *m/z*: [M+H]^+^ calculated for C_63_H_62_ClN_4_O_8_: 1037.4256, found: 1037.4230.

(*E*)-1-(1-(5-chloro-2-((3-cyanobenzyl)oxy)-4-((2-methyl-[1,1'-biphenyl]-3-yl)methoxy)benzyl)piperidine-2-carbonyl)-*N*-(4-((2-methoxy-4-(3-(4-methoxyphenyl)-3-oxoprop-1-en-1-yl)phenoxy)methyl)benzyl)piperidine-4-carboxamide (CP21). Yellow solid; mp: 125.3–126.3 ºC; ^1^H NMR (*d*6-DMSO) δ 8.34 (s, 1H), 8.17 (d, 2H, *J* = 8.7 Hz), 7.95 (s, 1H), 7.85 – 7.80 (m, 2H), 7.66 (d, 1H, *J* = 15.4 Hz), 7.58 – 7.43 (m, 4H), 7.42 – 7.27 (m, 7H), 7.25 – 7.17 (m, 3H), 7.09 (d, 3H, *J* = 9.1 Hz), 5.31 – 5.11 (m, 6H), 4.39 – 4.21 (m, 4H), 3.87 (d, 4H, *J* = 3.5 Hz), 3.49 (s, 2H), 3.01 – 2.85 (m, 6H), 2.66 (s, 2H), 2.35 (s, 2H), 2.23 (s, 3H), 2.08 – 1.94 (m, 4H), 1.72 – 1.63 (m, 3H), 1.42 (d, 5H, *J* = 28.1 Hz). ^13^C NMR (*d*6-DMSO) δ 173.33, 172.31, 163.46, 153.53, 150.37, 149.65, 142.64, 141.82, 139.14, 135.59, 132.82, 132.15, 131.23, 130.25, 130.16, 129.57, 128.60, 128.33, 127.73, 127.39, 126.00, 123.89, 114.49, 114.36, 113.61, 113.54, 111.91, 70.00, 56.17, 55.83, 53.07, 36.82, 33.10, 31.71, 31.29, 30.34, 29.47, 25.86, 16.10. HPLC: *t*_R_ 21.651 min, purity 97.154%. HRMS (ESI-Q-TOF) *m/z*: [M+H]^+^ calculated for C_66_H_66_ClN_4_O_8_ : 1077.4569, found: 1077.4574.

*In vitro* inhibition of PD-1/PD-L1. The PD-1/PD-L1 enzymatic assay (HTRF) was used to measure the potency of the target inhibitors to block the PD-1/PD-L1 interaction following the manufacturer’s instructions (*Cisbio*, catalog number: 64ICP01PEG&64ICP01PEH).

SPR, ITC, CD assay. Unless otherwise specified, all proteins used in the tests were purchased from Sino Biological Biotics Inc: human PD-L1 (catalog number: 10084-HNAH), murine PD-L1 (catalog number: 50010-M08H), human CXCL12 (Cat No: 13511-HNCE-2), murine CXCL12 (Cat No: 50025-MNAE-2). SPRi assay was carried out using PlexAray HT. In short, the concentration of h/mPD-L1 was diluted to 0.5 mg/mL with sterile water and was fixed to a bare gold-plated PlexAray nanocapture sensor chip via an amide bond. Then, **CP21** (various concentrations) was injected into the flow cell (30μL) by a nonpulsatile piston pump. ITC assay was conducted with a MicroCal PEAQ-ITC according to the manufacturer’s guidelines (Malvern Panalytical Ltd., UK). CD spectra was acquired from a Chirascan plus ACD. h/mPD-L1 and h/mCXCL12 were incubated with compound **CP21** or sterile water at 0 °C for 0.5 h, then CD wave scanning was carried out in the wavelength range of 180 ~ 260 nm at 0℃, with a step size of 1nm and a bandwidth of 2 nm.

Calcium flux assays. THP-1 cells were incubated with Fluo-4AM (Invitrogen, catalog number: F14201) calcium-dependent fluorophore for 0.5 h, washed and resuspended. Then, various concentrations of CXCL12 protein and compound **CP21** were added as specified in the text. Ca^2+^ flux was measured (Ex = 488 nm; Em = 520 nm).

Assessment of HepG2 cell mortality rate (Cellular). Firstly, HepG2 cells were stimulated with human IFN-γ (Peprotech, catalog number: 300-02) to express hPD-L1 stably. Jurkat T cells were stimulated with PHA-P (Invivogen, catalog number: inh-phap). Then, HepG2 (5×10^4^) and Jurkat T cells (5×10^4^) were added to the co-cultured model. Next, various concentrations of **CP21** were added. Finally, the Jurkat T cells were completely removed by washing the supernatant (200 μL) with cold PBS for 4 times. The mortality rate of HepG2 cells was determined by CCK-8 assay (Dojindo, CCK8, 500 test).

Pharmacokinetic Study in Male SD Rats. Male SD rats (300–360 g) were purchased from Liaoning Changsheng Biotechnology Co., Ltd. Diet. Blood samples (0.5 mL) were collected from the tail vein into heparinized 1.5 mL polythene tubes at 0.0833, 0.25, 0.5, 1, 1.5, 2, 4, 6, 8, 12, 24, 36, 48 h after oral (18 mg/kg) or intravenous (1 mg/kg) administration of compound **CP21**. Waters Acquity UPLC (Waters Corp., Milford, MA, USA) was used for the separation of **CP21** and internal standard compound. The effective separation of **CP21** and internal standard compounds was achieved on an Acquity UPLC BEH C18 column (50 × 2.1 mm, 1.7 μm, Waters Corporation, Milford, MA, USA) used at a column temperature of 40 °C. The mobile phase consisted of a mixture of solvent A (acetonitrile) and solvent B (formic acid/ ultrapure water, 1:1000, v/v). The linear gradient program for the mobile phase was set as follows: 0 min 10% A; 0.5 min 30% A; 1 min 95% A; 2 min 95% A; 2.3 min 10% A. The flow rate of the mobile phase was 0.4 mL/min, and the injection volume was 2 μL. The XEVO TQD triple quadrupole mass spectrometer was equipped with an electrospray ionization (ESI) source; multiple reaction monitoring (MRM) mode was selected for quantitation. The Mass Lynx 4.1 software (Waters Corp.) was used for data acquisition. Mass spectral data were obtained in positive electrospray mode (ESI+) in MRM mode.

*In vivo* efficacy study in a melanoma model and CT26 tumor model. Protocols for animal experiments (B16-F10 and CT-26 tumor model) were approved by the NIACEC (National Institutional Animal Care & Ethical Committee) at SMU (Southern Medical University). C57BL/6 male mice (6-8 weeks old) were purchased from Liaoning Changsheng Biosciences. Briefly, A total of 2×10^5^ B16-F10 melanoma cells or CT26 cells (1×10^6^) were inoculated into mouse (in the right flank) according to protocols of tumor transplantation studies in mice. **CP21** was dissolved in 30 % PEG-200, 5 % DMSO, and 65 % saline solution. Tumor volume was measured with a traceable electronic digital caliper (every 2 days).

Flow cytometry. Mouse organs were collected and tumor cells were mechanically isolated using a 40µm cell strainers. Cells were stained at 4°C for 30 min and flow cytometry (BD, USA) was performed using antibodies against the following targets (CD3, CD4, CD8, PD-L1) and isotype controls, all provided by BioLegend company: FITC anti-mouse CD3 Antibody (Cat No:100204, LOT: B249620), APC anti-mouse CD8a Antibody (Cat No:100711, LOT: B280032), Anti-mouse-PD-L1-PE Antibody (Cat#:124307, Lot: B284420), Rat IgG2b K Isotype Control PE (Cat#:400608, Lot: B156144).

Microscale thermophoresis assay. The concentration of blue labeled murine PD-L1 (Cat#:CJ88, Novoprotein Scientific Inc.) was kept constant, while the concentration of the unlabeled binding partner was varied. The unlabeled binding protein was titrated in 1:1 dilutions. **CP21** was diluted in MST-optimized buffer PBS. The murine PD-L1 was again added for 10 µl to the different concentrations of **CP21** (50 µmol-1.525 nmol) and fully mixed. For the measurement, **CP21** was filled into capillaries (NanoTemper technologies, Germany) and measurable after a 10 min equilibration at room temperature. The measurements were performed at auto LED power and medium MST power.

PBMC Killing Assay. Fresh PBMCs were isolated from healthy donors. MDA-MB 231 cells were seeded at a density of 2 × 10^3^ cells/well in the 96-well plates 12 h prior to the test. Different concentrations of compound **CP21** were added 2 h prior to the test. PBMCs (1×10^4^ cells/well) were stimulated with CD3/CD28/CD2 T cell activator (ImmunoCult, catalog no. 10970). Stimulated PBMCs were added to the MDA-MB 231 cells and test compound, and that mixture was then co-cultured at 37 °C for 12 h. Cell death was evaluated by the quantification of cell damage, which was evaluated by the release of LDH. The level of LDH released to the supernatant was detected by the LDH cytotoxicity assay detection kit (Beyotime) following the manufacturer’s instructions.

Determination of IFN-γ release. Briefly, Hep3B cells were engineered to stably express OS-8 (anti-CD3 single chain variable fragment) and human PD-L1 (hPD-L1). Fresh PBMCs were isolated from healthy donor by density gradient centrifugation. CD3 T cells were isolated from fresh PBMCs by EasySep™ Human T Cell Isolation Kit (negative selection, STEMCELL Technologies). Hep3B-OS8-hPDL1 cells were harvested and treated with 10 μg/mL mitomycin C at 37 ^o^C for 1.5h, and washed 4 times with PBS. Hep3B-OS8-hPDL1 and T cells (2.5x10^4^ in 50μL and 5x10^4^ in 100 μL complete media, respectively) were added to the 96-well plates, followed by the addition of 4x final concentration of test compound in 50 μL complete media according to the plate map, and co-culture at 37 ^o^C, 5% CO_2_ incubator for 72 h. The supernatants (150 μL) were harvested after 72 h of co-culture to determine IFN-γ levels by ELISA.

Statistical analysis. Prism software (version 5.0) was used for data analysis. Data were presented as mean ±SD unless otherwise stated. One-way variance (ANOVA) was used for calculating statistical significance (**P < 0.05).

Molecular docking. Glide 7.4 was used for visualization and molecular graphic manipulation. The crystal structures of PD-L1 and CXCL12 proteins were retrieved from PDB bank (4UAI and 5NIU).

Supplemental References

1 Cheng, B. *et al.* Discovery of Novel Resorcinol Dibenzyl Ethers Targeting the Programmed Cell Death-1/Programmed Cell Death-Ligand 1 Interaction as Potential Anticancer Agents. *J Med Chem* **63**, 8338-8358 (2020).

2 Cheng, B. *et al.* Discovery of Novel and Highly Potent Resorcinol Dibenzyl Ether-Based PD-1/PD-L1 Inhibitors with Improved Drug-like and Pharmacokinetic Properties for Cancer Treatment. *J Med Chem* **63**, 15946-15959 (2020).

Copies of ^1^H NMR, ^13^C NMR, HPLC, and HRMS spectra for compounds CP1–21.

































































**Supplementary Figures**


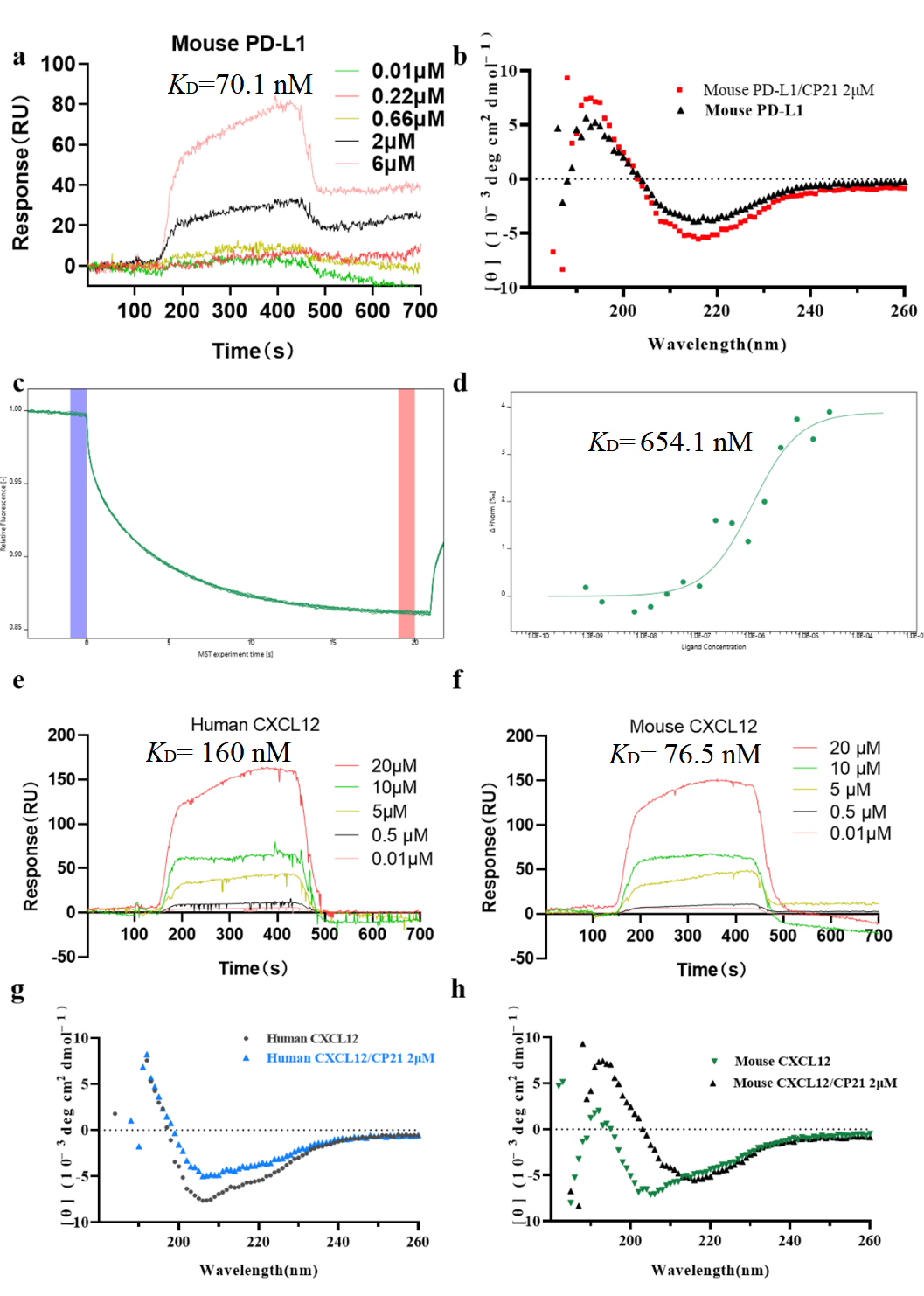


**Figure. S1** (a) The binding affinity of **CP21** to mPD-L1 as measured by SPR. (b) CD spectra of mPD-L1 in complex with or without **CP21**. (c) The MST traces of the binding affinity of **CP21** with mPD-L1. (d) The fitted curve of **CP21** with mPD-L1 as measured by MST. The binding affinity of **CP21** to hCXCL12 (e) and mCXCL12 (f) as measured by SPR. CD spectra of hCXCL12 (g) and mCXCL12 (h) in complex with or without **CP21**.


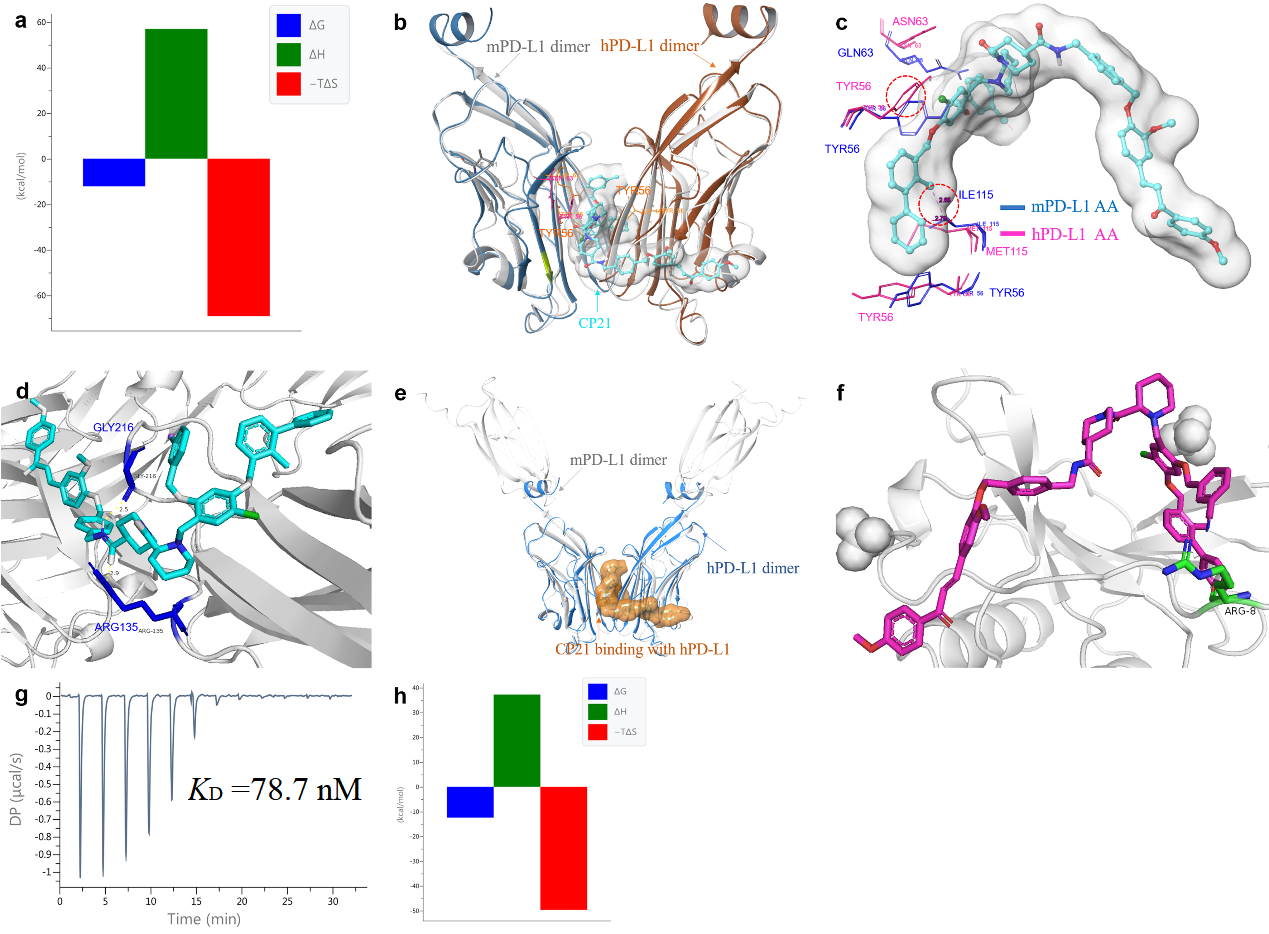


**Figure. S2** (a) ITC study for **CP21** in complex with PD-L1. (b) **CP21** in the binding domain of dimeric hPD-L1 (PDB: 6R3K). (c) The detailed binding interaction between compound **CP21** and dimeric hPD-L1. (d) CP21 in the binding domain of dimeric mPD-L1 (PDB: 6SRU). (e) Overlap of hPD-L1/CP21 (brown; PDB: 6R3K) and mPD-L1/CP21 (blue; PDB: 6SRU) structures. (f) Docking of **CP21** to human CXCL12 (PDB code: 4UAI). (g,h) ITC analysis of **CP21** with human CXCL12.


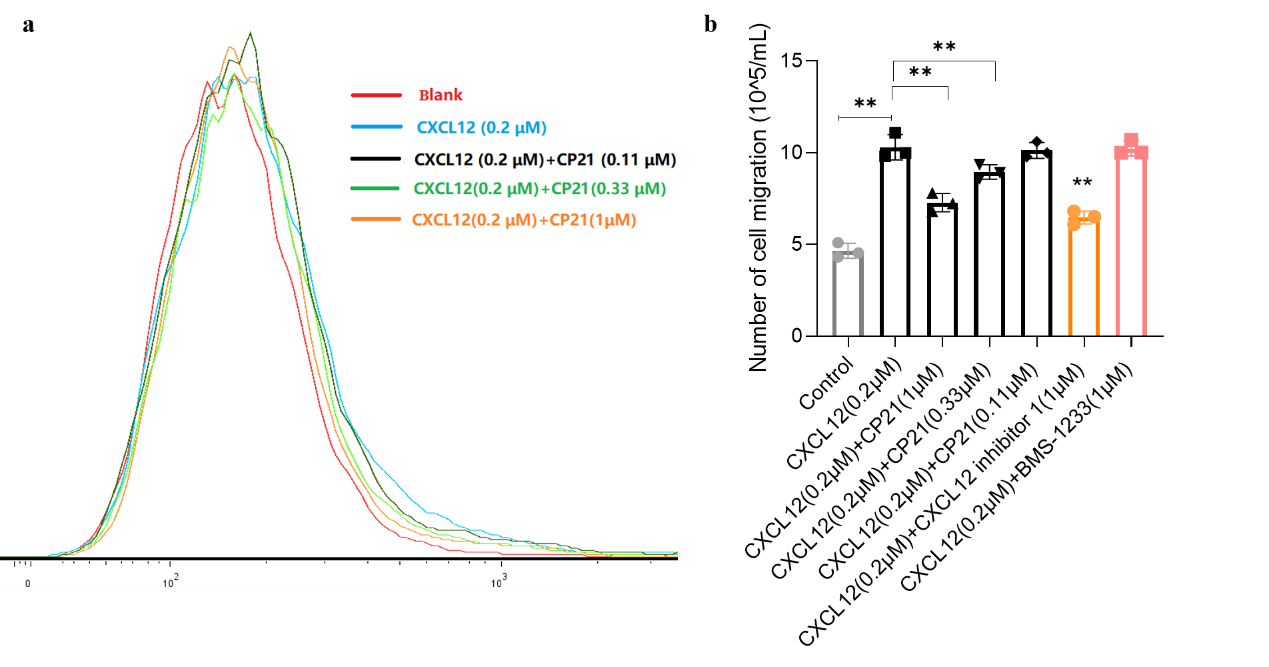


**Figure. S3** (a) Flow cytometry analysis of Ca^2+^ flux. (b) Transwell chemotaxis test in THP-1 cells.


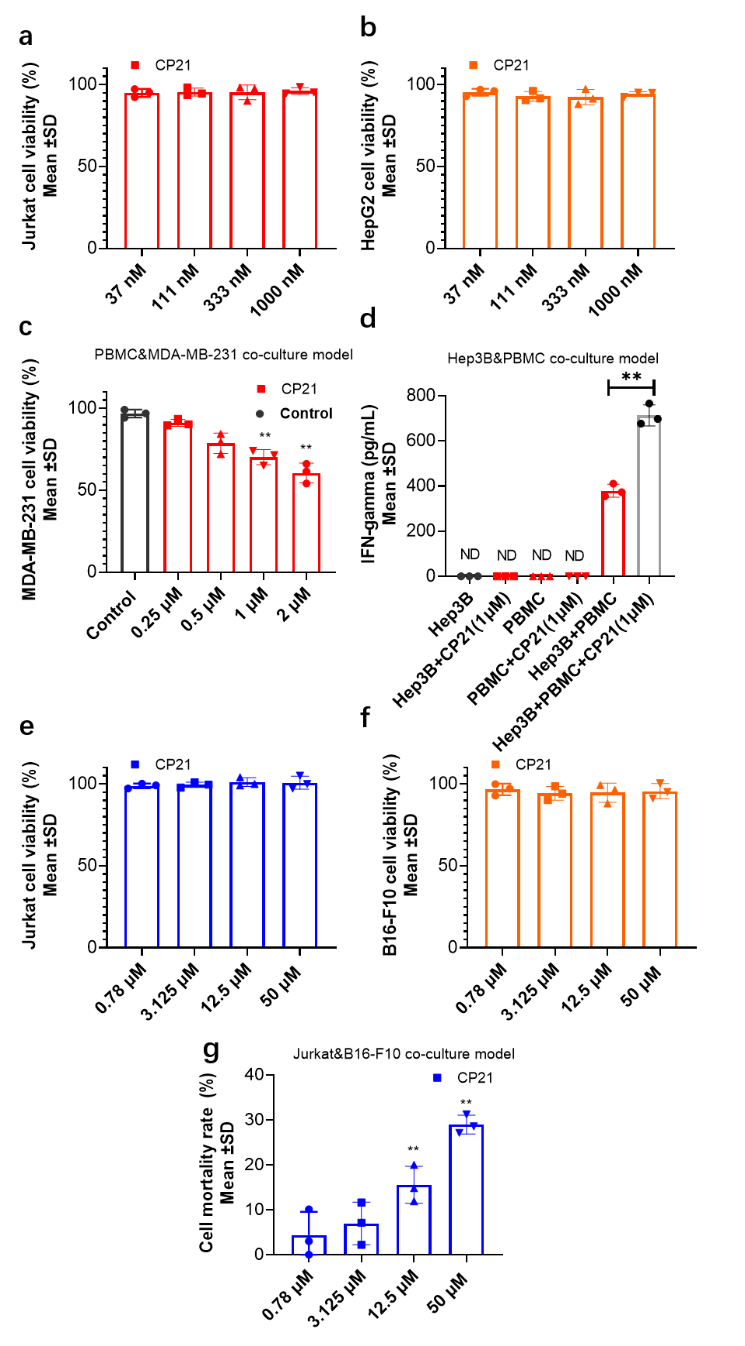


**Figure. S4** *In vitro* immune regulatory effects of CP21. Antiproliferative activities of CP21 against Jurakt cells (a), HepG2 cells (b). (c) Compound CP21 enhances the killing of MDA-MB 231 cells by PBMC. Tumor cell viability was detected by LDH release at MDA-MB 231 cells/PBMC (1:5). (d) Effects of CP21 on IFN-γ secretion by PBMC co-cultured with Hep 3B cells. Compound CP21 enhances the killing of B16-F10 cells by Jurkat cells. Antiproliferative activities of CP21 against Jurakt cells (e), B16-F10 cells (f), and in a B16-F10/Jurkat T cell co-culture model (g). All data are presented as means ± SD (**p < 0.05; n = 3).

**Figure. S5** Body weight change in mice.


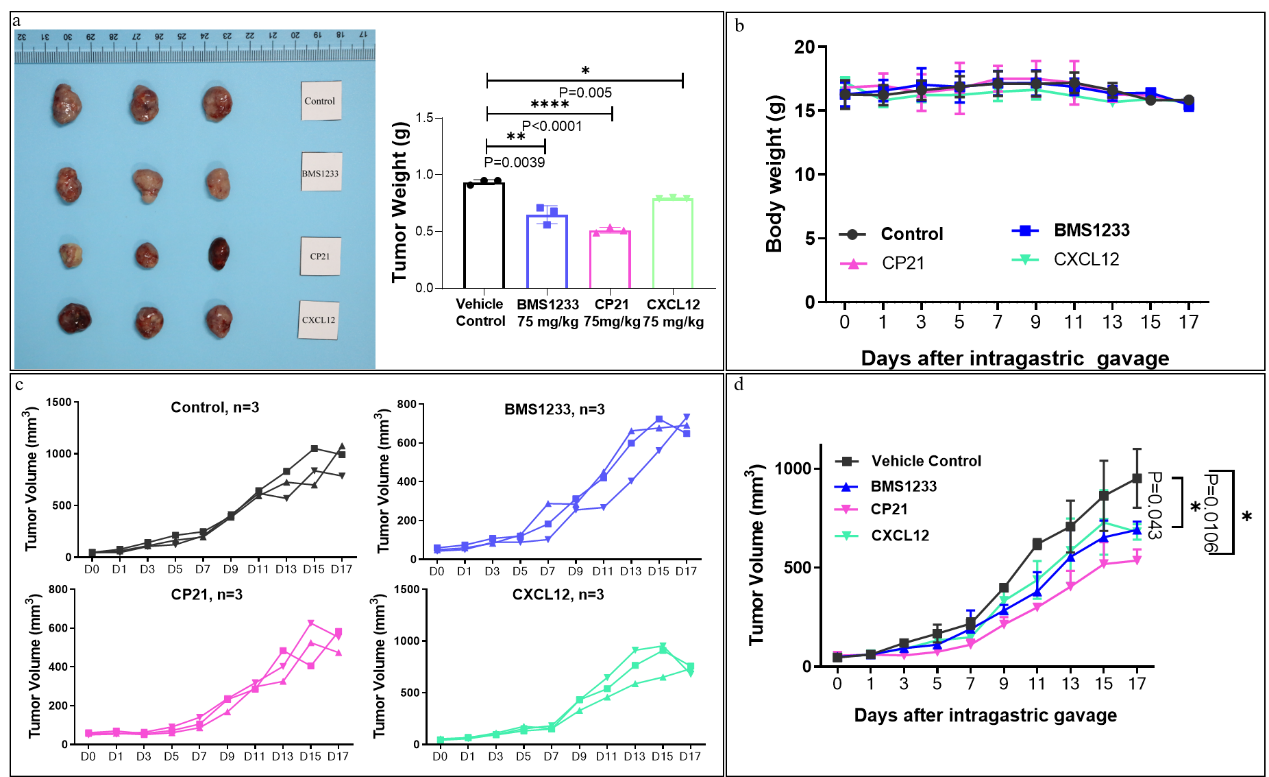


**Figure. S6** *In vivo* anti-tumor efficacy of CP21 in a CT26 tumor model in mice. Mice were treated with vehicle control, BMS1233 (75 mg/kg), CP21 (75 mg/kg), and CXCL12 inhibitor (75mg/kg per day) for 17 days consecutively. Then, sacrificed and tumors weights and volumes were measured. (A) Images of tumors and weight of tumors; (B) changes of body weights of mice during treatment; (C) Individual tumor growth curves over time (D) changes of tumor volume during treatment. *P < 0.05, **P < 0.01, and ****P < 0.0001 (n=3).

**
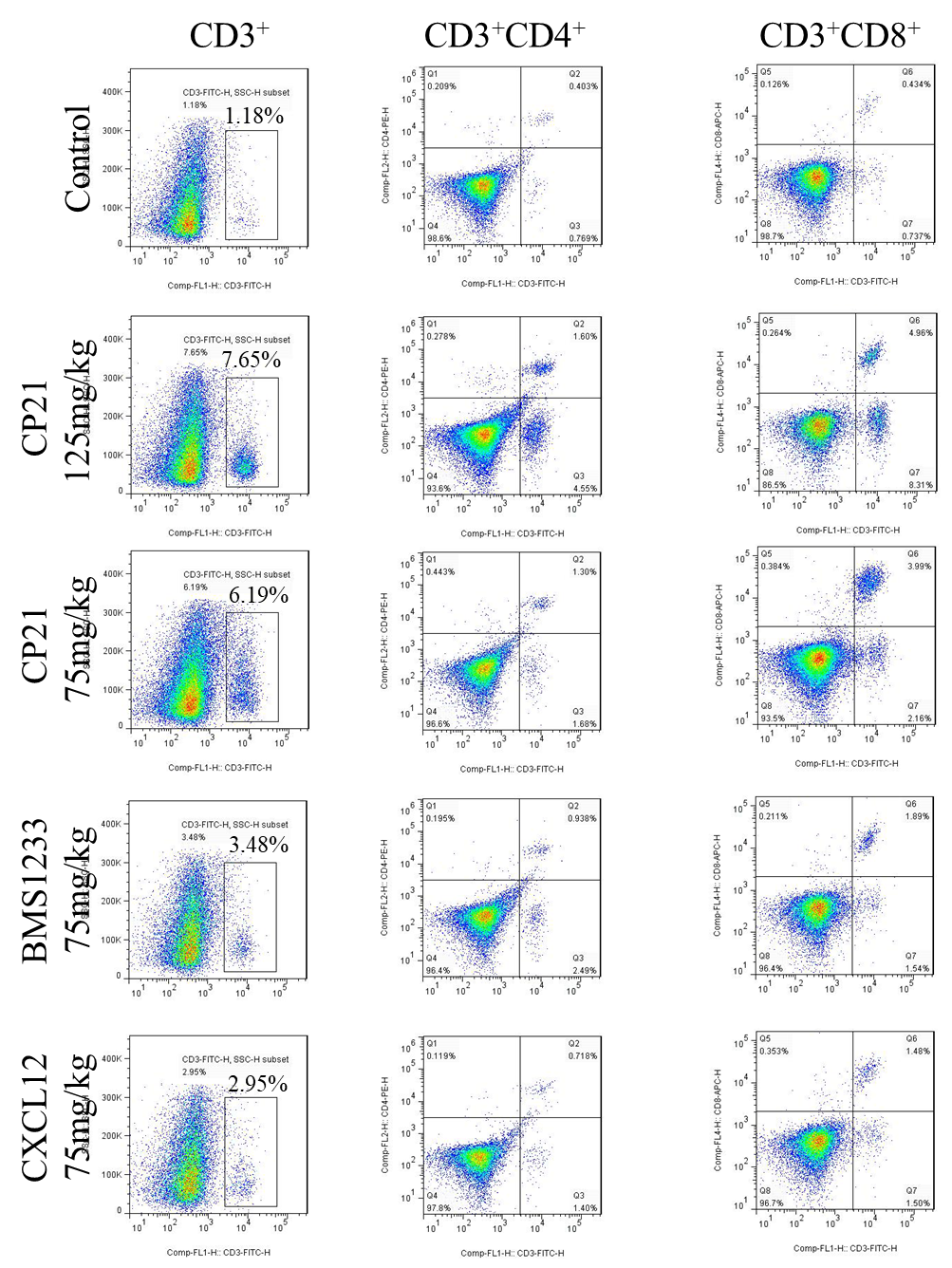
**

**Figure. S7** Representative images for the percentages of TILs in different treatment groups.

**
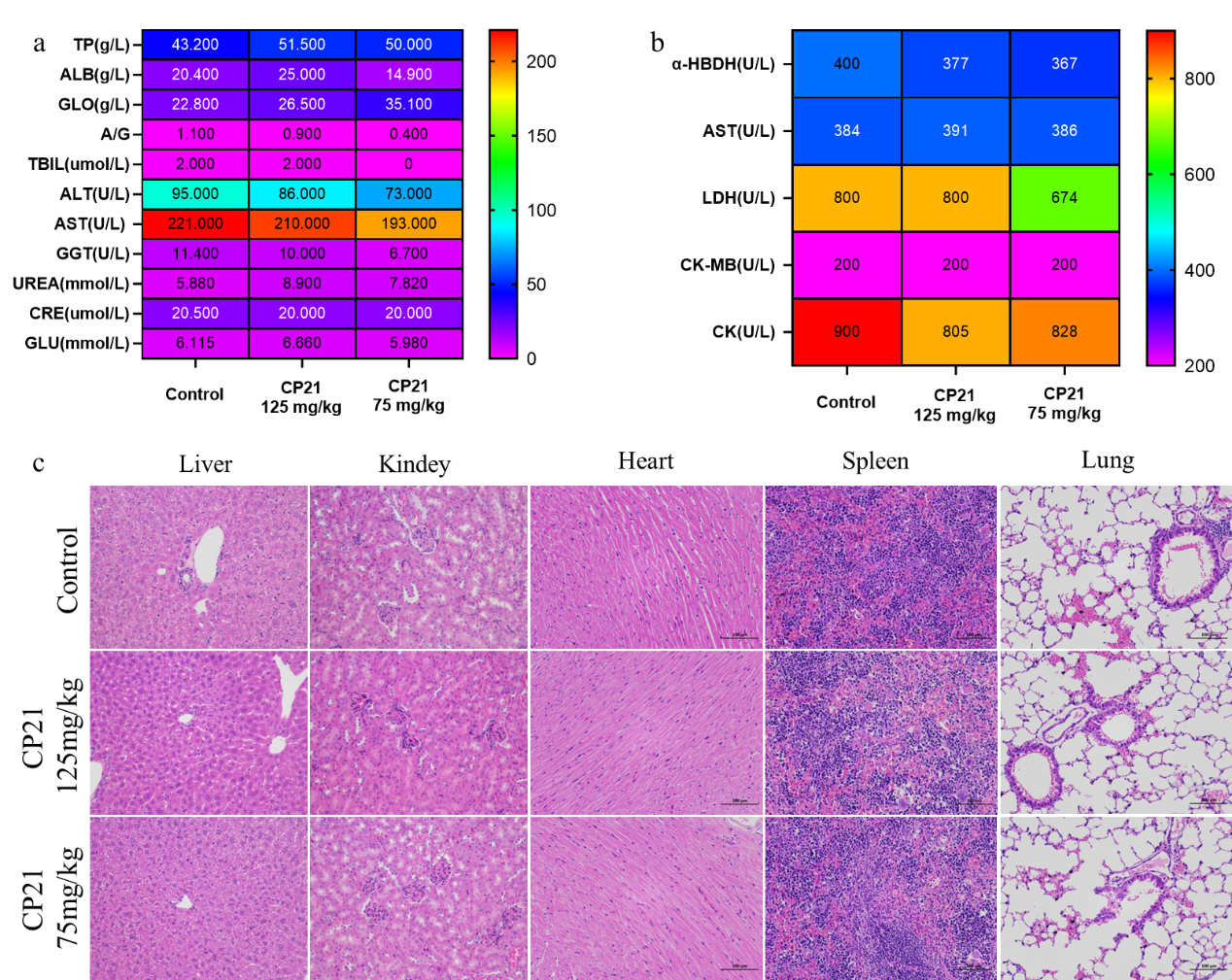
**

**Figure. S8** (a) Profiling of serum biochemistry for the function of liver and kidney. (b) creatine kinase isoenzymes assay. (c) H&E staining of mice organs.

**Supplementary Tables**

**Table S1** Anti-PD-1/PD-L1 potency of bifunctional inhibitors **CP1–21**.

| **Structure** | | | | | |
| --- | --- | --- | --- | --- | --- |
|  | | |  | | |
|  | | |  | | |
|  | | | | | |
| **ID** | **R_2_** | **IC_50_ (nM)** | **ID** | **R_2_** | **IC_50_ (nM)** |
| CP1 |  | > 1000 | CP12 |  | > 1000 |
| CP2 | H | > 1000 | CP13 |  | > 1000 |
| CP3 |  | > 1000 | CP14 | H | > 1000 |
| CP4 |  | > 1000 | CP15 |  | > 1000 |
| CP5 |  | > 1000 | CP16 |  | > 1000 |
| CP6 | H | > 1000 | CP17 | H | 126.5 |
| CP7 |  | > 1000 | CP18 |  | 107.3 |
| CP8 |  | > 1000 | CP19 |  | >1000 |
| CP9 |  | 100.7 | CP20 |  | 88.5 |
| CP10 | H | > 1000 | CP21 |  | 78.6 |
| CP11 |  | 870 | BMS-202 69.7 | | |

The synthetic route for target compounds **CP1-21** is shown in **Scheme S1**. Firstly, various PD-L1 inhibitors (BMS-1233, CH-1, CH-11, BMS-8, NP19) were synthesized according to the reported routes (**Scheme S1A-C**).^1^ Then, various CXCL12 inhibitor 1-linker conjugates were coupled with appropriate PD-L1 inhibitors to generate target compounds **CP1-21** by a condensation reaction (**Scheme S1D**).

Scheme S1. Synthesis of CP1–21^α^

The inhibitory potency of the newly synthesized compounds (**CP1-21**) against PD-1/PD-L1 was assessed via an HTRF assay.^2^ As detailed in **Table S1**, compounds **CP1-85** [with BMS-8 (IC_50_ =146 nM) as the PD-L1-targeting warhead], **CP6-8** [with CH-1 (IC_50_ =1 μM) as the PD-L1-targeting warhead] and **CP13-16** [with CH-11 (IC_50_ =1 μM) as the PD-L1-targeting warhead] showed no/little inhibitory activity with IC_50_ >1 µM. While compounds **CP9-12**, with NP19 (discovered by us before, IC_50_ =12.5 nM) as the warhead^3^, exhibited improved anti-PD-1/PD-L1 activities than that of the aforementioned compounds (**CP1-8, 13-16**) with IC_50_s in the range of 100.7 ~ 870 nM. The last series of bifunctional inhibitors **CP17-21**, with BMS-1233 (IC_50_ =14.5 nM) as the PD-L1-targeting warhead, displayed better PD-1/PD-L1-inhibitory activities with IC_50_s ranging from 78.6 nM to 126.5 nM. Among these compounds, **CP21** exhibited the highest anti-PD-1/PD-L1 potency (IC_50_ = 78.6 nM). Based on the above results, we found that the PD-L1-targeting warhead is critical for anti-PD-1/PD-L1 potency, the higher activity of the PD-L1-targeting warhead, the better the PD-L1-inhibitory activity of bifunctional molecules.

**Table S2** *In vivo* Pharmacokinetic Properties of **CP21** (Mean ± SD).

| **Pharmacokinetic parameters** | **I.V. administration**  **(1 mg/kg)** | **P.O. administration**  **(18 mg/kg)** |
| --- | --- | --- |
| **AUC_(0–t)_ (****ng/mL·h)** | 2032.5 ± 11.4 | 127.5 ± 4.8 |
| **AUC_(0–∞)_ (ng/mL·h)** | 2138.0 ± 18.3 | 127.9 ± 4.6 |
| **MRT_(0–t)_ (h)** | 11.3 ± 0.4 | 5.9 ± 0.2 |
| **MRT_(0–∞)_ (h)** | 14.0 ± 0.5 | 6.0 ± 0.2 |
| ***t*_1/2_ (h)** | 12.4 ± 0.3 | 2.2 ± 0.8 |
| **T_max_ (h)** | 0.083 | 6.3 ±0.8 |
| **CL (L/h/kg)** | 0.5 ± 0.1 | 140.8 ± 5.0 |
| **Vz (L/kg)** | 8.4 ± 0.2 | 467.4 ± 184.8 |
| ***C*_max_ (ng/mL)** | 325.5 ± 58.1 | 20.5 ± 3.1 |
